# Energizing Intention to Visit Rural Destinations: How Social Media Disposition and Social Media Use Boost Tourism Through Information Publicity

**DOI:** 10.3389/fpsyg.2021.782461

**Published:** 2021-11-23

**Authors:** Yunfeng Shang, Khalid Mehmood, Yaser Iftikhar, Atif Aziz, Xuedan Tao, Liting Shi

**Affiliations:** ^1^School of Hospitality, Zhejiang Yuexiu University, Shaoxing, China; ^2^School of Economics and Management, Tongji University, Shanghai, China; ^3^AFPGMI, National University of Medical Sciences, Rawalpindi, Pakistan; ^4^Karachi Institute of Economics and Technology, Karachi, Pakistan; ^5^School of Arts and Media, Tongji University, Shanghai, China

**Keywords:** information publicity, social media disposition, theory of planned behavior, social media use, subjective norm, intention to visit

## Abstract

This study aimed to examine the impact of information publicity on the intention of tourists to visit rural destinations in developing countries. Based on the theory of planned behavior (TPB), we examined the indirect effect of information publicity on intention to visit *via* subjective norms and further investigated the moderating effect of social media disposition and social media use. The study used data from a time-lagged design with three waves which supported the hypothesized model. The findings revealed that information publicity has an influence on the intention of tourists to visit through the mediating effect of subjective norms. Moreover, the social media disposition strengthened the relationship between information publicity and subjective norms. Furthermore, social media use positively moderated the relationship between subjective norms and intention to visit. Besides the core TPB constructs, the added variables indeed exerted a substantial impact on the visit intention of tourists. The study contributed to the tourism-related literature on social media and the practical implications were discussed.

## Introduction

How does the intention to visit rural tourist destinations be developed and strengthened to expedite economic activity that may contribute to the growth and development of the world? Tourism is a high potential industry, and the potential of rural tourism is yet to be tapped. In 2019, World Tourism and Travel Council anticipated a contribution of $39.8 billion within a decade. Even for a developing country like Pakistan, tourism is a high potential sector, which can substantially contribute to economic growth. The government in the country has also vowed to promote tourism in anticipation of a $1 trillion contribution to the economy by 2025. Sustainable tourism is being considered a tool for economic growth and development (Rafiq, 2019). Its role in wealth formation, livelihood, and income generation is well recognized (Li et al., 2018; Merli et al., 2019). Tourism is related to development, as the rising number of new destinations indicates, thus its role in socio-economic advancement is multifold (The World Bank, 2019; Zaman et al., 2021). Owing to its immense potential and contribution to socio-economic development, in the last decade, tourism literature encompassed studies on the visit and revisit intention of tourists (Tajeddini et al., 2021). Intention to visit play a key role in the growth and sustainability of tourism (Ngoc and Trinh, 2015). Intention to visit has been considered as a major determinant of growth and survival of the tourism business because it helps in forecasting marketing expenses (Kim et al., 2013), profitability (Alves et al., 2019), and sustainability of tourism business (Stylos et al., 2017). Furthermore, the trade-off between attracting and retaining the costs of tourists determines the competitive advantage (Chiu et al., 2012; Kim et al., 2013; Abbasi et al., 2021), which is also a function of tourists visit intentions.

Few studies are found on rural tourism, which attempts to reveal the decision-making processes regarding rural destinations. Rural tourism is relatively less explored, but the potential area in developing countries for tourism purposes. It can be exploited to enhance the potential of the tourism sector in countries like Pakistan (Chaudhary, 2004). As rural destinations are known for simplicity, natural and organic environment less disturbed by industrialization where green fields and forests are preserved for long, the cost of maintaining such destinations may be as low as zero. Thus, giving very cheap alternatives to highly expensive urban tourist destinations, beaches, and mountainous tourist destinations. Therefore, in this study, we have focused on the intention to visit rural destinations.

The intention to visit is influenced by several factors (García-Fernández et al., 2018) because the decision to travel is an intricate decision-making process that is affected by information publicity, social media disposition, social media use, subjective norms, attitude, and perceived behavioral control (PBC) in addition to several others (Han and Kim, 2010; Bianchi et al., 2017). Among others, social media disposition and social media use can be two conspicuous factors that may strongly influence intention to visit. Their role as facilitators of interaction between information publicity, social norms, and intention to visit may unfold several subtleties of the visit decision-making process.

Nowadays, social media and social networking sites have revolutionized the ways people receive information and news (Lou and Yuan, 2019; Khan, 2021; Mujahid and Mubarik, 2021). Even in developing countries like Pakistan, the use of social media sites like Facebook, Twitter, WhatsApp, YouTube, Flicker, and Instagram (Aftab and Khan, 2019) is high with respect to tourism-related information sharing. This bunch of social media networking sites points to the diversity and intensity of information people receive, comprising thousands of commercials, forums, blogs, vlogs, etc. (Ganguly, 2015; Lou and Yuan, 2019; Majeed et al., 2020). Young people, especially millennials and youngers, have become habitual in using social media in their day-to-day lives (Gottfried and Shearer, 2016). It has become inevitable for them to use social media to address their information needs.

One aspect of media that also affects social media use is social media disposition, which refers to social media richness and social media authenticity. Social media is a major source of information for tourists; thus, its disposition is expected to play facilitating role in realizing the impact of information being publicized in terms of reinforced social norms. The social media richness measured in terms of feedback capability, number of channels, sources of information, and language variety (Daft and Lengel, 1986) determines the ability of media to convey complex messages (Cao et al., 2021). The selection of media is made to suit the characteristics of the message and the audience (Cao et al., 2021). Another aspect of social media disposition is content trustworthiness, which may affect social norms. As social media is an open forum for all to share information, the problems related to content trustworthiness become severe. Social media users show less reliance on information from sources with a dubious disposition (Hovland and Weiss, 1951; Lou and Yuan, 2019). In such circumstances, disassociation of information from a source produces a better impact than in situations otherwise. Owing to the importance of social media disposition as a facilitator between information publicity and subjective norms, we have included it as a key moderator in our study.

Advancement in information and communication technology has resulted in a paradigm shift in the tourism business (Milano et al., 2011; Pesonen, 2011). The use of social media and social networking services is no more limited to personal use instead, people collect information, disseminate information, share feedbacks and experiences that influence the decisions of others. As tourism is an information-intensive industry, people seek information before deciding on a destination to ensure greater value for money and an enriched experience. Thus, social media play a central role in tourism decisions nowadays (Hays et al., 2012). Literature shows that there is less research on the reinforcement role of social media use intensity between subjective norms and the intention to visit of tourists.

**FIGURE 1:**
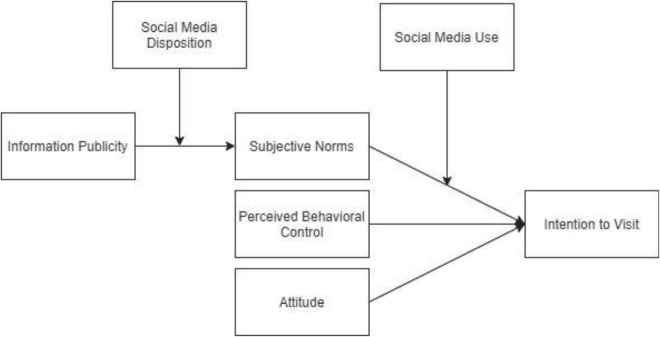
Conceptual model of the study.

It is evident that the theory of planned behavior (TPB) has wide application in research for understanding consumer intentions in terms of subjective norms, attitude, and PBC (Meng and Choi, 2019; Hasan et al., 2020; Meng and Cui, 2020; Soliman, 2021). In tourism-related research studies, TPB has been applied to unfold the intention of tourists to visit and revisit destinations (Hasan et al., 2020; Joo et al., 2020; Abbasi et al., 2021; Soliman, 2021). We intend to apply TPB to study the impact of social media on the intention to visit of rural tourists. This study uses TPB to examine the mechanism through which social media disposition and social media use moderate relationships between information publicity, subjective norms, and intention to visit the destinations of rural tourists. Therefore, on the one hand, this study will contribute to the extant literature on TPB; on the other hand, it is expected to contribute to the literature on tourism research, visit intention of tourists, and factors underlying the decision-making processes about tourism. Practical insights from this study will enable businesses, managers, and regulatory bodies to focus their efforts on the elements driving the intention to visit of tourists. Hence, this study will have a wide range of practical and managerial implications. The introduction of social media disposition and social media use intensity as facilitating mechanisms is unique to this study. Thus, this study will also contribute to the literature on social media research. The conceptual model of this study is illustrated in Figure 1.

## Literature Review and Hypothesis Development

### Theory of Planned Behavior

The TPB was developed by Ajzen (1991), which aimed at understanding human behavior. As we know, decision-making is a complex process, TPB explains that when there is the intention to engage in a behavior, three distinct factors can be identified, which play a key role in developing intention and shaping behavior. Namely, attitude, subjective norms, and PBC, which embody internal (attitude) and external (subjective norms) forces that determine the extent to which an individual is likely to engage in a behavior subject to the perception of an individual about his control on engaging in that behavior (PBC), respectively (Joo et al., 2020). Many research studies have applied and validated the TPB model in various research settings (Quintal et al., 2010; Jalilvand and Samiei, 2012). Quintal et al. (2010) studied intent of visit regarding Asian tourism destination in Aussie respondents using the TPB model. Their study also tested the effect of perceived risk and perceived uncertainty on subjective norms, attitude, and PBC. As it is encouraged by Ajzen (1991) to test TPB for the decision-making process regarding tourism, hence researchers apply TPB widely in tourism studies to unfold the underlying decision-making processes. A study on wine tourism conducted by Sparks (2007) used TPB to analyze revisit intention within a year of the first visit. They found that except for the emotional attitude, all other component factors in TPB had an effect on revisit intention. Another TPB-based study on the impact of electronic word of mouth on destination choice for tourism indicated that word of mouth has become digitalized and significant (Jalilvand and Samiei, 2012; Hussain et al., 2020; Fan and Dong, 2021). The study was conducted in Isfahan, Iran, and it was found that word of mouth impacts the subjective norm, PBC, and attitude. With the rise in the use of TPB in tourism studies, its application to study sustainable tourism also rose. Kuo and Dai (2012) studied intention to visit low carbon tourism sites in the future. Several other studies use TBP in extended form (Ashraf et al., 2019; Meng and Choi, 2019) or in a framework with other theories (Lee and Jan, 2018) to study tourism-related issues. Furthermore, community-based tourism was studied by Eom and Han (2019). This study attempts to analyze the intention to visit rural tourism places using TPB as the overarching mechanism to explain the role of social media disposition and social media use intensity in connection with information publicity, subjective norms, attitude, and PBC.

### Information Publicity and Subjective Norms

In this study, by information, we imply all the information related to rural tourism. The behavior of an individual is determined by the information possessed by that individual (Pettigrew et al., 2001); thus, lack of information results in the absence of related behavior. Despite the importance of tourism in socio-economic development and governmental support for promoting tourism in Pakistan, still tourist enthusiasm to visit rural destinations in low (Adeyinka-Ojo and Khoo-Lattimore, 2013; Wen and Huang, 2019). This lower rural tourism is partly attributed to the lack of information on rural tourist destinations (Mihailović and Moric, 2012). Tourists equipped with information on tourism destinations, hotels, restaurants, transportation services, and other tourism services are more likely to visit rural destinations (Li et al., 2019).

Subjective norms in our study refer to the pressure felt by individuals due to norms of significant ones in their surroundings, making them exhibit or refrain from certain behavior (Manning, 2011). In other words, if people find significant people in their family, relatives, leaders, and colleagues expecting them to engage in the desired behavior, then people are more likely to adopt that behavior (Carmeli and Schaubroeck, 2007). Thus, if family members, colleagues, and leaders prefer rural tourist destinations and want them to prefer those too, it is more likely to influence people.

Relationship between information publicity and subjective norms have been discussed in various context (Wang et al., 2018, 2019). In the context of intention to participate in voting, Bergan et al. (2021) studied the influence of information publicity and subjective norms in university students. Their study showed that information publicized to students and subjective norms significantly affects their intention to participate in voting provided that they have already overcome registration barriers. It is evident that a lack of information discounts on the influence of subjective norms. Providing information on rural tourism may subsidize the information costs of not preferring rural tourist destinations. The motivational dimension of subjective norms can be observed through the subjective norms lens. In this context, we hypothesize the following:

H1: Information publicity significantly influences subjective norms.

### Subjective Norms and Intention to Visit

Subjective norms are an integral component of the TPB model, which influences behavioral intentions. These are functions of the belief of people regarding what they feel others who are important to them would do in a specific context under consideration (Kessler, 2013). In other words, it refers to the feeling of social pressure by someone due to actions and opinions of the important ones to her or him (Bianchi et al., 2017). The referent others may be friends, family, colleagues, and any other groups of people relevant to the situation. Subjective norms are a function of the interpretation of individuals regarding the referent interpretation of others when it comes to behavior and inspiration to give outlook in liking of their beliefs and expectation (Ajzen and Fishbein, 1977). Hence it can be referred to as communal pressure to enforce a behavior as the person will seek approval. In marketing and tourism literature, subjective norms have been recognized as an important driver of behavioral intentions (Quintal et al., 2015; Hasan et al., 2020). In tourism-related literature, it is empirically proven that destination choices are influenced by the opinions of tourists about whether important others will approve or not the specific vacation destination (Nicoletta and Servidio, 2012). Moreover, it has been found that the distance between the destination of tourists and their home country neither stimulates subjective norms nor expedites their development or affects their influence on intentions (Abbasi et al., 2021). To test the influence of subjective norms on intention to visit a rural destination in our setting, we hypothesized.

H2: Subjective norm significantly influences intention to visit.

### The Mediating Role of Subjective Norms

A distinguishing feature of TPB from other behavioral theories is its allocation of mediating role to behavioral intent between the effects of attitude and subjective norms on behavior. All other factors affect behavioral intentions via subjective norms and attitudes. It is established that intentions are the outcome of various internal and external incentives and facilitators (Hornik et al., 1995). TPB can help to establish that information publicity and subjective norms drive intentions to visit such that subjective norms mediate the relationship between information publicity and intention to visit. Information publicity being a robust environmental antecedent of consumer behavior adds to the effects of subjective norms. Lack of information about certain aspects of tourism may cause a fall in motivation to visit. Visitors unaware of the existence of the destinations of rural tourists, tourism services, transportation facilities, reviews of tourists, experience sharing channels, and other relevant information tourists seek before picking destination are likely to lack enthusiasm (Guiver and Stanford, 2014) compared to people having necessary information (Cole, 2006).

Building on TPB, it can be suggested that information publicity is a source of enhanced subjective norms, thereby fostering social pressure felt by a person in response to subjective norms (H1). In turn, subjective norms foster the intention to visit rural destinations, i.e., the important ones in the surrounding of tourists may influence their intention to visit a particular destination because they endorse it by their experience sharing, recommendations, comments, and by sharing necessary information they have about the tourist destination, its path, travel services, hospitality services, etc. (H2). Therefore, we speculate that information publicity indirectly influences intention to visit rural destinations through subjective norms. Thus, we hypothesize the following:

H3: Subjective norm positively mediates the relationship between information publicity and intention to visit.

### Behavioral Control and Intention to Visit

According to Ajzen (1991), the concept of PBC is considered as an antecedent of behavior and intentions. Furthermore, it is the perception of difficulty or ease in performing a behavior (Kastenholz et al., 2018), i.e., PBC refers to the opinion of an individual of whether they are capable of doing the behavior. PBC primarily considers the perceptions of individuals regarding how effectively dealing with aspects that may permit or limit a particular behavior. Abbasi et al. (2021) asserted in this regard that perceptions of exertion toward a behavior would have a negative impact on the intention to execute that behavior. PBC is determined by the perception of an individual regarding the availability or lack of resources and chances for executing a certain behavior, as well as the perceived significance of such opportunities and resources for doing such behavior (Mark and Christopher, 1998). Prior studies on destination choice indicate that PBC has a significant and positive influence on the behavioral intentions of individuals (Boley et al., 2018). Hence, ability, time, and resources all play a role in forecasting the intention of an individual to engage in a behavior. Therefore, we propose the following hypothesis.

H4: Perceived behavioral control significantly influences intention to visit.

### Attitude and Intention to Visit

Drawing insight from TPB, attitude can be referred to as the sentiments of an individual related to the consequences of a behavior (Soliman, 2021). Attitudes can be defined as prominent beliefs which can be influenced by observation, a piece of information, or an inferential process (Sharma et al., 2021). Hence, people form favorable or unfordable attitudes about the consequences of behavior based on their beliefs. Attitudes, in turn, influence intentions to engage in the behavior. Prior studies in the field of tourism reveal a substantial positive link between attitudes toward a destination and intentions to visit (Khasawneh and Alfandi, 2019; Nguyen Viet et al., 2020). As though the beliefs of tourists regarding the consequences of traveling to a destination are likely to change (i.e., financial, time), still we may inference that their attitudes will have no differential impact on their intentions to visit despite the attitude being based on those beliefs. This leads to the following hypothesis.

H5: Attitude significantly influences intention to visit.

### The Moderating Role of Social Media Disposition

People engaged in the promotion of tourist destinations might have felt strongly about the importance of social media. The majority of rural tourist enterprises are too small to afford extensive promotion (Kladou and Mavragani, 2015); on the other hand, smaller tourist destinations can also benefit from social media advertising. As a result, social media promotion is particularly crucial for rural tourism. It is simple to identify promotions conducted through social media. Tourists use social media to get information and share trip details. Amaro et al. (2016) conducted research on social media in tourism by clustering approach. They classified the study into six categories, including social media and tourist behavior. They discovered that the amount of social media-related tourism research rose rapidly after 2010. Schuckert et al. (2015) analyzed tourism-related social media studies from 2007 to 2011. Their findings revealed that social media research was performed on both supply and demand sides. Many studies have been done on the demand side to see how social media affects individuals who are trying to make travel arrangements. Also, there have been several studies on the supply side about social media as a strategy. Though social media researches for tourism have mainly focused on consumer behavior and satisfaction since 2010, several new ideas, such as big data, digital marketing, and online reviews, have lately been embraced for tourist studies (Lin et al., 2020).

Social media disposition is viewed as a positive and negative influence (Bergan et al., 2021) on the outcomes of individuals, such as intentions and behaviors. In relation to subjective norms, which address the motivation dimension of individuals, information publicity is not a very powerful inducer of changes in intentions of individuals thus, researchers prefer to explain the weakness of relation in terms of intervening variables. For example, it has been argued that mere information publicity cannot incite the behavior because information may be distorted and manipulated in the transmission process, thus losing its effectiveness. Supportive findings of Bernstad (2014) reported the ineffectiveness of written information dissemination to generate intended behavior. The study of Wang et al. (2018) elaborated that those inconsistent research results were the outcome of poor information quality. In other words, its information quality or in broader terms, social media disposition that determines the relationship between information publicity and the related behavior (Mickaël, 2014; Wang et al., 2018). Social media disposition is one of the strongest factors likely to strengthen the positive influence of information publicity. Scholars have researched social media marketing at times in order to give valuable insights for tourism advertisers. Zeng and Gerritsen (2014) examined social media usage for marketing by small and medium companies. The findings revealed that enterprises either contained much worthless content or were considerably less developed. Hays et al. (2012) discussed the proper approach to perform social marketing after studying the social marketing tactics of ten of the most famous countries for overseas tourists. Kavoura (2014) regarded the importance of social media in the tourism sector as establishing an online tourist community and stressed the significance of marketing from this perspective.

Our study speculated that the influence of information publicity on the intentions of tourists depends on social media disposition. Social media disposition is a composite of media richness and content trustworthiness. Thus, if the information receivers perceive that information provided is precise, reliable, pertinent, timely, comprehensible, and inclusive (Mickaël, 2014; Wang et al., 2018) they are more like to act as desired (Chang, 2013). Nevertheless, on the contrary, less credible and inaccurate information will weaken the relationship between information publicity and specific behavior (Lee et al., 2007; Mickaël, 2014). It means that if tourists think that information publicized to them is pertinent, useful, precise, reliable, and comprehensive, they will be tended to feel more subjective norms to develop the intention to visit (Mickaël, 2014; Zhang et al., 2017; Wang et al., 2018). In other words, if the information is perceived to be from a rich and trustworthy source, the impact of information publicity on the visit intention of tourists will be stronger (Mickaël, 2014; Zhang et al., 2017; Wang et al., 2018). Therefore, it can be speculated that social media disposition positively moderates the relationship between information publicity and the perceived subjective norms of tourists to visit the tourist sites. Thus, we hypothesize the following:

H6: Social media disposition moderates the relationship between information publicity and subjective norms such that the relationship is stronger for tourists with a higher level of social media disposition than with a lower level of media disposition.

This study is carried out to determine the bright side of social media on tourism. Social media plays a significant role in the online tourism sector when individuals make vacation schedules (Munar and Jacobsen, 2013; Molinillo et al., 2018). Kwok and Yu (2013) researched to determine the impact of Twitter and Facebook on the appeal of tourism websites. According to their findings, Facebook had a significant influence in raising the number of visits to tourism websites. Munar and Jacobsen (2014) investigated the impact of social media on the process of arranging a vacation. It was discovered that the use of social media had an effect on the original plans of tourists. People utilized social media the most after their vacations were finished to share their travel experiences with others. Travel information found on social media and provided by other users was deemed more trustworthy by social media users than information obtained from other sources (Fotis et al., 2012). Narangajavana et al. (2019) investigated the connection between tourist perceptions and social media use. Tourists who used social media more regularly viewed user-generated material more often.

Furthermore, obtaining user-generated material affected the overall image of user-generated information, which in turn influenced the expectations of visitors. Overall, people are using social media to socialize and make connections, stay up to date on events, and seek facts (Liu et al., 2018). The most prevalent social media usage of tourists is to share tour photographs and experiences (Fotis et al., 2012). As the relevance of social media usage in tourism rises, scholars aim to understand the motivations underlying social media usage (Fox and Rooney, 2015). According to Ahmed et al. (2019), motives for sharing online material include personal cognition and individual action, as well as community-related motivation. Hausmann et al. (2018) utilized social media data to investigate whether predicting the preference for environmentally friendly tourism locations was feasible. The social media data revealed specific features. It was established that tourism-related content published on social media is substantial enough to be utilized in place of traditional surveys to offer insight.

H7: Social media use moderates the relationship between subjective norms and intention to visit such that the relationship is stronger for visitors with a higher level of social media use than with a lower level of social media use.

## Materials and Methods

For the purpose of this study, we data collected purposively from residents of Lahore, Pakistan, who visited some rural destinations. To achieve the objectives of this study, we targeted tourists who were Facebook, Twitter, and WhatsApp users following the patterns of previous studies by Feng et al. (2021) for similar research objectives. Pakistan is fifth in the ranking of the most populated countries in the world whereas Punjab is the most populous province in the country with plenty of rural tourist destinations in the south and middle of the province. Lahore is in the north of the province and people usually visit several rural places for vacations. As most of the population is in cosmopolitan cities, respondents from Lahore, the capital city of Punjab represent an adequate sample and prior studies results provide evidence for generalizability of the findings to the whole country from the studies conducted on samples from cosmopolitan cities (He et al., 2020). Before the formal investigation, the respondents were informed that their participation was anonymous and they would receive a reward of Rs. 200 after completing the investigation.

The time-lagged method was utilized in this study to gather data at three-time intervals. The time-lagged technique is common in modern research since it allows researchers to perform many surveys for a specific study (Khan et al., 2020; Ali et al., 2021; Bahadur and Ali, 2021; Khan, 2021a,b; Mehmood et al., 2021a,b; Ullah et al., 2021; Yu et al., 2021). Researchers may collect data from several sources in this manner, reducing the possibility of common source bias. Furthermore, this method enables data collection at various time intervals, minimizing the likelihood of common method bias. Similarly, this method enables participants to analyze and take action their behavior before recording their final response (Detert and Edmondson, 2011). Three waves of data collection at 2-week intervals were conducted to avoid common method bias (Podsakoff et al., 2003; Ali et al., 2020; Cao et al., 2020). In the first-wave survey (T1), a survey was shared with 700 respondents. A total of 620 completed questionnaires were returned (88.5% response rate). Respondents were asked to complete the demographic information and the questions regarding information publicity, social media disposition, PBC, and attitude. The second-wave survey (T2) took place 2 weeks later; the 620 respondents were asked to report their subjective norms and social media use. The number of returned usable responses was 540 (87.09% response rate). In the third-wave survey (T3), respondents were asked to complete questions regarding the intention to visit, with 503 valid responses received (93.1% response rate). The study sample comprised 380 (75.5%) males and 123 (24.5%) females. The average age of the respondents was 29 years, 33.8% of respondents had a bachelor’s degree, and 39.2% had a Master’s degree. The average income of the respondent is almost Rs. 400,000.

### Measures

The questionnaires utilized scales adapted from previous studies for the constructs under study and the demographic variables. All study variables were assessed utilizing a five-point Likert scale (1 = strongly disagree; 5 = strongly agree). The original questionnaire was developed in English and then translated into Urdu using the back-translation method (Brislin, 1980). To assess information publicity (α = 0.953), a 7-item scale was used, drawn from Wang et al. (2018). The sample item: “I think the relevant information publicity for rural tourism is important.” To measure social media disposition (α = 0.931), we used an 8-item scale developed by Cao et al. (2021). The sample item: “When the social network site provides rich and varied communication and response.” We used a 4-item scale to measure subjective norms (α = 0.936), developed by Meng and Choi (2016). The sample item: “Most people who are important to me think I should go to rural tourism sites.” A four-item scale was used to measure PBC (α = 0.809), adapted from Meng and Choi (2016). The sample item: “I am capable of going to rural tourism sites.” A four-item scale was adapted from Kassem (2003) to measure attitude (α = 0.914). The sample item: “Going to rural tourism sites is enjoyable.” We used a four-item scale to measure social media use (α = 0.865), developed by Narangajavana et al. (2017). The sample item: “Using social network sites is part of my daily activity.” To assess intention to visit (α = 0.915), a 4-item scale was used, drawn from Meng and Choi (2016). The sample item: “I intend to travel to rural tourism sites in the next 1 year.”

## Results

### Descriptive Statistics

The correlation matrix, standard deviations, means, and the reliabilities of the variables of the study are displayed in Table 1. Correlations among the variables of study provide initial support for hypothesis testing.

**TABLE 1:** Descriptive statistics, reliabilities, and correlation matrix.

	Mean	SD	CR	AVE	1	2	3	4	5	6	7	8	9	10	11
1. Age	2.700	1.10	–	–	–										
2. Gender	0.760	0.430	–	–	0.04	–									
3. Education	2.660	0.875	–	–	0.07	0.13**	–								
4. Income	2.770	1.124	–	–	0.10*	0.17**	0.70**	–							
5. Information publicity	2.863	1.510	0.924	0.732	0.04	0.18**	0.15**	0.23**	(0.953)						
6. Media disposition	3.110	1.267	0.894	0.589	0.12**	0.02	0.02	0.08	0.15**	(0.931)					
7. Subjective norm	2.845	1.375	0.935	0.782	0.02	0.01	0.02	0.07	0.45**	0.36**	(0.936)				
8. Perceived behavioral control	4.028	0.703	0.811	0.520	0.06	0.10*	0.09*	0.04	0.02	0.04	0.04	(0.809)			
9. Attitude	2.564	1.089	0.914	0.727	0.02	0.01	0.11*	0.01	0.03	0.15**	0.07	0.05	(0.914)		
10. Social media use	3.953	0.795	0.857	0.601	0.02	0.02	0.01	0.06	0.03	0.06	0.01	0.53**	0.04	(0.865)	
11. Intention to visit	3.616	1.321	0.908	0.712	0.04	0.01	0.10*	0.12**	0.31**	0.04	0.36**	0.16**	0.17**	0.13**	(0.915)

Notes: **p < 0.01, *p < 0.05; N = 503.

### Confirmatory Factor Analysis

Confirmatory factor analysis (CFA) was performed by using AMOS 24 to examine the convergent and discriminant validity of the studied variables by following the recommendation of Anderson and Gerbing (1988). The cut-off criteria of Hu and Bentler (1999; i.e., χ2/df less than 2; CFI greater than 0.9, and RMSEA less than 0.07) were used to test convergent and discriminant validity. We include multiple item variables to conduct CFA on individual-level data to confirm the validity of data. The results of the CFA analysis are displayed in Table 2. The baseline model test results show that the seven-factor was the good fit with the data (χ2/df = 599.11/409 = 1.465; CFI = 0.985; TLI = 0.982; and RMSEA = 0.03) compared with alternative models. As displayed in Table 3, the factor loadings (λ: cut-off criteria greater than 0.6 and *p* < 0.001) were all above 0.638, and all items observed showed significant loadings on their related factors. We also examined the average variance extracted (AVE; cut-off criteria > 0.5) and composite reliability (CR; cut-off criteria > 0.8), leading to convergent validity being supported (see Table 3). Together, the model proposed was deemed suitable for hypotheses testing.

**TABLE 2:** Confirmatory factor analysis.

Model	χ^2^	*df*	χ*^2^/df*	Δχ^2^ (Δ*df*)	TLI	CFI	RMSEA
Seven-factor model: baseline model	599.11	409	1.465		0.982	0.985	0.030
Six-factor model: combining IP, SMD, SN, SMIU	3230.18	424	7.618	2631.06 (15)	0.869	0.832	0.147
Five-factor model: combining IP, SN, PBC, AT	3478.81	425	8.185	2879.7 (16)	0.771	0.772	0.210
Three-factor model: combining IP, SMU, SN, PBC, AT	3627.75	427	8.495	3028.64 (18)	0.644	0.709	0.260
Four-factor model: combining IP, SMD, SN, PBC, AT	4342.65	429	10.122	3743.54 (20)	0.610	0.687	0.267
Two-factor model: combining SMD, SMU, SN, PBC, AT, ITV	4460.12	429	10.396	3861.01 (20)	0.514	0.583	0.279
One-factor model: combining all into one factor	4671.62	430	10.864	4072.51 (21)	0.617	0.668	0.340

*Notes: IP, Information publicity; SMD, Social Media deposition; SN, Subjective Norm; PBC, Perceived behavioral control; AT, Attitude; SMU, Social media use; ITV, Intention to visit; TLI, Tucker–Lewis index; CFI, Comparative fit index; and RMSEA, Root-mean-square error of approximation.*

**TABLE 3:** Variable reliabilities and convergent validity.

Variables	Items code	λ	CR	AVE
Information publicity (IP), (Time-1)	IP1-IP7	0.832–0.935	0.924	0.732
Social media deposition (SMD), (Time-1)	MD1-MD8	0.748–0.908	0.894	0.589
Subjective norm (SN), (Time-2)	SN1-SN3	0.821–0.981	0.935	0.782
Perceived behavioral control (PBC), (Time-1)	PBC1-PBC4	0.638–0.794	0.811	0.520
Attitude (AT), (Time-1)	A1-A4	0.819–0.878	0.914	0.727
Social media use (SMU), (Time-2)	SMIU1-SMIU4	0.680–0.832	0.857	0.601
Intention to visit (ITV), (Time-3)	ITV1-ITV4	0.793–0.908	0.908	0.712

*Notes: All factor loadings are significant at (p < 0.001), N = 503; λ = factor loadings, AVE, average variance extracted; CR, composite reliabilities.*

### Structural Model and Hypothesis Testing

**FIGURE 2:**
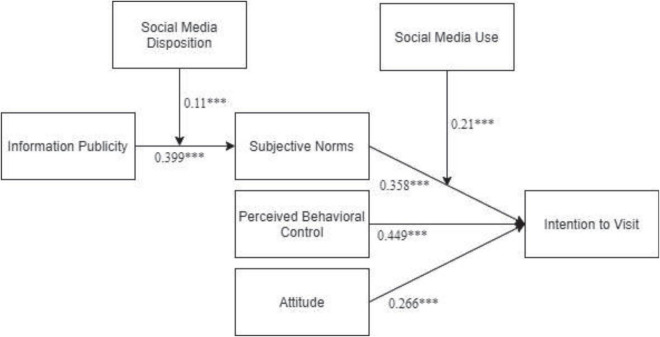
Path analysis results; ***P < 0.001.

We analyzed the conceptual model by using the SEM approach, and in prior studies, this approach [SEM: using Analysis of Moment Structures (AMOS) 24.0] has been extensively used (Khan and Khan, 2021). Fit indices showed that the proposed model has an adequate fit (χ2/df = 273.619/184 = 1.487; CFI = 0.981; TLI = 0.987; and RMSEA = 0.031). H1 proposed that information publicity significantly influences subjective norms. The path analysis (Figure 2) reveals that information publicity significantly influences subjective norms (β = 0.399, *t* = 8.736, *p* < 0.001), which supports H1. H2 proposed that subjective norms significantly influence intention to visit. The path analysis reveals that subjective norms significantly influence intention to visit (β = 0.358, *t* = 6.408, *p* < 0.001), which supports H2. The indirect effect of information publicity on intention to visit through subjective norms [0.102, 95% CI = (0.0694,0.148)] was significant, and hence, hypothesis 3 was supported. H4 proposed that PBC significantly influences intention to visit. Figure 2 results reveals that PBC significantly influences intention to visit (β = 0.449, *t* = 3.891, *p* < 0.001), which supports H4. The results (Table 2) also reveal that attitude significantly influences intention to visit (β = 0.266, *t* = 4.266, *p* = 0.001), which supports H5.

**FIGURE 3:**
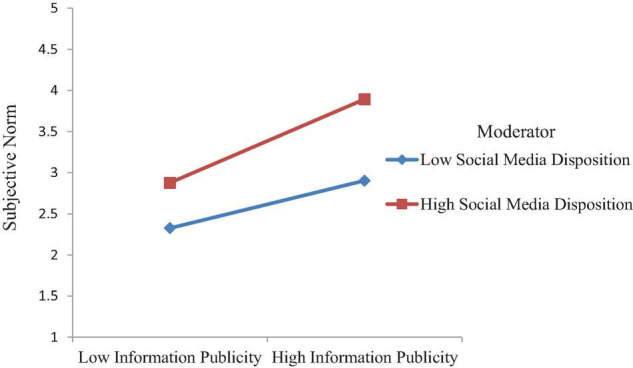
Interactive effects of information publicity and social media disposition on subjective norm.

This study further proposed that social media disposition enhances the positive effect of information publicity on the subjective norms of tourists. The relationship is stronger for tourists with a higher level of social media disposition than with a lower level of media disposition. Accordingly, the findings (Figure 2) reveal that there is a significant interaction between information publicity and social media disposition on the subjective norms (β = 0.11, *t* = 4.097, *p* < 0.001), which supports H6. We also assessed moderating effect pattern visually following Aiken and West (1991) to plot the interaction. Figure 3 shows that the relationship between information publicity and subjective norms becomes stronger at higher levels of social media disposition, supporting H6.

**FIGURE 4:**
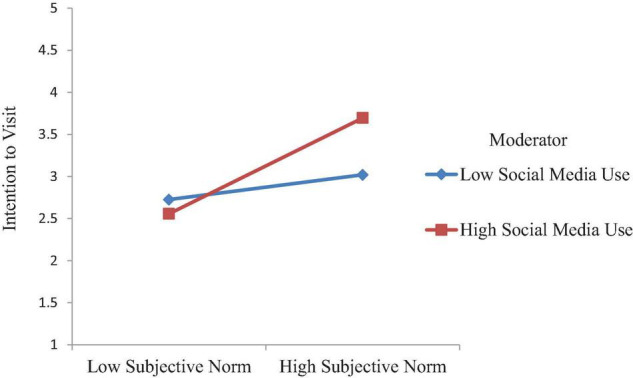
Interactive effects of subjective norm and social media use on intention to visit.

For H7, we proposed that social media use enhances the positive effect of subjective norms on the intentions to visit of tourists, such that relationship is stronger for tourists with higher-level social media use than with lower level. Accordingly, the findings (Figure 2) reveal that there is a significant interaction between subjective norms and social media use on the intentions to visit tourists (β = 0.21, *t* = 4.117, *p* < 0.001), which supports H7. We also assessed moderating effect pattern visually following Aiken and West (1991) to plot the interaction. Figure 4 shows that the relationship between subjective norms and intentions to visit tourists becomes stronger at higher levels of social media use, supporting H7.

## Discussion and Conclusion

Grounded on TPB, the findings of this study confirmed the hypotheses related to the relationships among information publicity, social media disposition, social media use, and intention to visit. As the results from the time-lagged design, it is found that subjective norms mediate the information publicity-intention to visit relationship. Therefore, we had theoretically expanded the TPB model by Ajzen (1991) in conformance with his criteria for extending the theory. With respect to overall tourist behavior, this study is a step forward in expanding and developing the theory in the existing tourist behavior literature (Han and Kim, 2010). This study, in particular, contributes valuable insights about the visit intention of tourists while broadening the TPB model by including social media disposition and social media use. The findings showed that all proposed hypotheses about direct relationships were validated. The findings revealed that PBC influences the intent of the visitor. Tourism is not something that people can live without. In addition, planning a vacation needs both time and money. Sustainable tourism, in particular, may not be the first option for individuals who have never traveled before. Subjective norms had a considerably stronger influence on the intentions of travelers to visit rural areas. The same result was demonstrated in prior studies that used the TPB model to other tourism destinations (Jalilvand and Samiei, 2012; Ashraf et al., 2019). This suggests that in order to boost popularity, it is critical to creating a positive public image of visiting tourism sites. Moreover, social media disposition strengthens the relationship between information publicity. Additionally, social media use also strengthens the relationship between subjective norms and intention to visit, such that the relationship is stronger when social media use is at a high level than for those with a low level of social media use.

### Theoretical and Practical Implications

The findings of this study offer several meaningful theoretical contributions to the tourism-related literature in many ways. First, this study employed the TPB to investigate how social media disposition and social media use boost the visit intentions of tourists. To the best of our knowledge, this is the first research of its nature that has expanded the TPB model to explain the variables influencing the visit intentions of tourists. Prior studies on tourism in Pakistan have mainly focused on tourist destination image (Nazir et al., 2021), but no study has explored the factors that influence the visit intention of tourists by using social media disposition and social media use.

Second, to extend the TPB and improve the tourism-related body of knowledge, this study used information publicity, social media disposition, and social media use. The conceptual model of this study, which included the variables of information publicity, social media disposition, and social media use, enables elucidating a significant proportion of the visit intentions of tourists and demonstrates the compatibility of the model. The addition of these variables broadens the existing tourism-related literature on TPB because no other study has given a holistic model that incorporates all examined factors to demonstrate their influence on tourist visit intentions.

Third, the findings of this study, particularly regarding the antecedents of the TPB, such as subjective norm and attitude, add to the existing body of knowledge. These findings contradict the findings of previous research (Abbasi et al., 2021), particularly in the context of tourism in South-Asian countries. Pakistan is a developing South-Asian country that is culturally and expressively distinct from the developed countries where previous studies were conducted. Thus, the implementation of the TPB in the South-Asian region throws fresh light on the model of the TPB and opens up new avenues for future scholars to study how these aspects may be studied further in future research.

Finally, because this study focuses on the tourism industry in a developing nation such as Pakistan, it contributes to and broadens the existing knowledge about the tourism industry from a relative viewpoint. Most prior tourism studies sampled developed countries. Due to cultural variations between countries, findings from one country may not apply to other countries, which may have distinct preferences and expectations based on common cultural or societal norms (Huang and Crotts, 2019).

This study was practically beneficial for local and global social media advertisers and managers seeking to reach tourists and influence their intentions to visit. Especially insights from this study could prove beneficial for rural tourism destinations managers, travel services, travel consultancy providers, and related hospitality services providers. Understanding and expanding on the knowledge gained from this study would allow the people related to the tourism industry of the country to strategize to get a strategic advantage in this lucrative industry.

First, to gain an edge, tourist destinations managers, restaurants, hotels, travel agency services, tour advisers, tour consultants, and other tourism-related services providers should develop strategies for information publicity using social media to boost the intentions of visitors to visit while focusing on the satisfaction of tourists by giving more value for money and better service to increase their intention to visit. Identifying the information of tourists needs to help them choose rural destinations over other choices is vital for effectively strategizing. Tourists need information on several aspects of tour-related questions while planning and choosing a tour destination. In this regard, information on the availability of travel services, hospitality services, scenic views, adventure supports, security, safety, and price of various services may be important for different tourists subjectively. In the context of rural destinations, availability of amenities of life, cultural food, unique spots for locally famous food, spots for recreation, security of routes, cultural values of local people, folk tales, etc., may be an essential part of the information necessary to trigger visit intention. Advertisement of such locations, services, and reviews about the satisfaction and picture sharing on social media of travelers can be considered a vital source of social norm building, which may help visitors choose rural locations over other options.

Second, tourism-related services providers are recommended to establish an online presence and urge their travel customers to share their vacation photographs on social media sites, which induces benign envy in consumers (Latif et al., 2020), triggering travel desire. The significant presence of tourism-related services providers from any destination and any destination contributes to the subjective norm development. The tourism-related services providers, especially brands known to customers, may act as significant ones that develop pressure on tourists to choose a particular location supported and recommended by those service providers, which they trust or with whom they had a great experience in the past. Thus, the feeling of pressure because of subjective norms, in turn, forms the intention to visit. Therefore, it can be asserted that rural destinations and tourism service providers for rural destinations should not ignore the importance of their online presence on social media.

Third, this study finding revealed that social media support rural tourism-related decision-making processes of tourists. On the one hand, the disposition of social media is key to convince tourists to choose a rural destination over others. Enriched content, factual information from reliable channels, and candid reviews from social media users may change the stats of the industry in the country. On the other hand, when tourists take rural tourism into account, the societal environment and the situations of individuals really contribute to encouraging their visiting intentions. Therefore, it may be stated that marketers should prioritize information linked to subjective standards to support the growth of sustainable tourism. Marketing practices should foster social norms and emphasize the functional benefits of rural tourism rather than just the attractions of the individual tourist sites. Sustainable tourism growth might help to promote the focus of marketers on fostering the sharing of tourism experiences on social media. Developments may be made to enable and promote the act of sharing through social media (e.g., attractive photographic spots and sites). Marketers may also arrange certain events for tourists, including activities to share on social media, such as awards for uploading scenic posts. Events that can go beyond personal enjoyment to sustained societal benefits might promote more strong subjective norms through social media disposition.

### Limitations and Future Research

This study employed time-lagged data, which generally controls the common method bias (Podsakoff et al., 2012), so common source bias and common method bias were not big concerns. Despite these advantages, our study has several limitations. First, the data were collected from a single type of industry located in the Lahore city of Pakistan, hence, it cannot be generalized to other industries and national contexts. Second, although our data were collected from tourists, which could be considered as a more than adequate setting for the tourism sector in terms of studying social media use for the attraction of tourists and further studies may broaden this focus to include different geographical areas, cultures, or times to increase the generalizability of the model. Western and Eastern tourists perceive tourism-related decision-making differently (Müller et al., 2021), and the comparison process differs depending on individualist/collectivist culture (Mehmood and Hussain, 2017a,b; Mehmood and Li, 2018; Li et al., 2019; Mehmood et al., 2019, 2020; Jabeen et al., 2020; Latif et al., 2020). As a result, future cross-cultural research on Western and Eastern cultures would be fascinating.

## Data Availability Statement

The raw data supporting the conclusions of this article will be made available by the authors, without undue reservation.

## Author Contributions

KM, YI, and YS conceived the idea and helped in writing the introduction and literature review. YI and AA collected the data. KM and YI wrote the discussion, conclusion, and implications of the study. LS helped in writing the Literature Review. All authors have read and approved the final manuscript.

## Conflict of Interest

The authors declare that the research was conducted in the absence of any commercial or financial relationships that could be construed as a potential conflict of interest.

## Publisher’s Note

All claims expressed in this article are solely those of the authors and do not necessarily represent those of their affiliated organizations, or those of the publisher, the editors and the reviewers. Any product that may be evaluated in this article, or claim that may be made by its manufacturer, is not guaranteed or endorsed by the publisher.
